# TSP50 in Neural Stem Cells Regulates Aging‐Related Cognitive Decline and Neuroinflammation by Altering the Gut Microbiota

**DOI:** 10.1111/acel.70188

**Published:** 2025-08-05

**Authors:** Xiaoli Li, Yuecong Chen, Zhuoyi Gao, Xiaoling Liu, Zhenbo Song, Feng Gao, Shuyue Wang, Chunlei Yu, Luguo Sun, Yanxin Huang, Lihua Zheng, Guannan Wang, Ying Sun, Jiawei Li, Xiaoguang Yang, Yongli Bao

**Affiliations:** ^1^ National Engineering Laboratory for Druggable Gene and Protein Screening, Northeast Normal University Changchun China; ^2^ International Joint Research Center of Stem Cell Bank, Ministry of Science and Technology Northeast Normal University Changchun China; ^3^ The Key Laboratory of Molecular Epigenetics of Ministry of Education, Northeast Normal University Changchun Jilin China

**Keywords:** aging, gut microbiota, neuroinflammation, TSP50

## Abstract

Aging is a process of gradual decline in physical and cognitive function and is a major risk factor for mortality. Despite the increasing number of relevant studies, the mechanisms regulating the aging process have not been fully elucidated. Genetic factors have long been recognized as key factors in controlling the rate of aging. Testes‐specific protease 50 (TSP50) has been shown to be involved in the regulation of embryonic development and intestinal homeostasis, but its role in the regulation of aging remains unclear. Here, we showed that TSP50 expression was reduced in the hippocampus of both aged humans and mice. TSP50 deficiency in neural stem cells (NSCs) drove accelerated aging in mice, characterized by exacerbated age‐related cognitive impairments and significantly elevated neuroinflammation. Notably, aged mice with NSCs‐specific knockout of TSP50 exhibited impaired intestinal mucosal barriers, dysbiosis of gut microbiota, and a marked reduction in the production of short‐chain fatty acids (SCFAs). Restoring gut microbial ecology using fecal microbiota transplantation (FMT) and overexpressing TSP50 successfully alleviated aging‐associated cognitive decline and neuroinflammation. Taken together, our study suggests that TSP50 plays a critical role in the aging process and identifies gut microbiota as a pivotal mediator of TSP50's influence on age‐related cognitive decline and neuroinflammation. These findings highlight the potential therapeutic value of targeting TSP50 and gut microbiota for aging, offering insights into aging mechanisms and interventions for aging‐related neurodegenerative diseases.

## Graphical abstract

TSP50 expression declines in the hippocampus of aged humans and mice. TSP50 deficiency in NSCs accelerates mouse aging, causing cognitive impairments and neuroinflammation. Restoring gut microbiota via FMT and TSP50 overexpression alleviates these phenotypes. Our study shows TSP50's critical role in aging and gut microbiota as a key mediator.

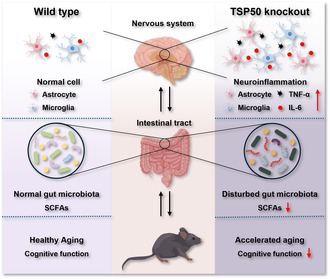

## 1 Introduction

Aging is a gradual and complex physiological process (Guo et al. 2022). It usually affects the physiological functions of the brain, leading to cognitive decline and increased neuroinflammation, accompanied by the occurrence of various neurodegenerative diseases, such as Parkinson's disease (PD), Huntington's disease (HD), and Alzheimer's disease (AD) (Bhat et al. 2024; Rim et al. 2024). According to a report published by the World Health Organization, the global population over 60 years old will reach 1.4 billion by 2030 and close to 2.1 billion by 2050, accounting for 21.3% of the world's population (World Health Organization 2024). The continuing rise in the proportion of the elderly population poses a significant global challenge (Hua et al. 2024). Therefore, it would be very helpful to clarify the mechanisms underlying the progression of aging. Changes in the gut environment have been shown to be closely related to aging (Martel et al. 2022). The intestinal mucosal barrier, composed of the mucus layer and tight junctions of intestinal epithelial cells, facilitates the absorption of beneficial nutrients and prevents harmful substances and pathogens from passing through the epithelial cells (Barbara et al. 2021). Aging causes damage to the intestinal mucosal barrier, which in turn leads to systemic inflammation (Zheng et al. 2022). In addition, alterations in gut microbiota are closely associated with the aging process (Ma et al. 2021). With aging, the composition of gut microbiota changes, which may influence an individual's overall health status, accelerate the aging process, or increase the risk of disease (O'Toole and Jeffery 2015). Healthy aging can be promoted by regulating gut microbiota (Jang et al. 2024). Studies have shown that transplanting gut microbiota from young mice into old mice can reduce cognitive impairment, reverse differences in hippocampal metabolites, reduce peripheral and brain inflammation, and thus delay brain aging (Heijtz et al. 2021). Furthermore, it has been reported that probiotic supplements improve physiological function and cognitive ability in aged mice by regulating gut microbiota (Kim et al. 2021). Thus, gut microbiota plays a crucial role in brain function, especially in brain aging, and genes involved in the regulation of the intestinal mucosal barrier and gut microbiota colonization may play a pivotal role in aging.

Genetic pathways have long been recognized as key factors in controlling the rate of aging. Numerous studies have shown that many genes can affect the aging process by regulating gut microbiota, such as ApoE, LCN2, and ATP11b (Chen et al. 2022; Liu et al. 2022; Singh et al. 2020). TSP50 is a serine hydrolase that is expressed in a variety of tissues, including the brain and intestine (Yuan et al. 1999). Studies have indicated that TSP50 plays a significant role in regulating embryonic development and neurodevelopment (Ai et al. 2018). Moreover, our previous study has shown that TSP50 is essential for maintaining intestinal homeostasis and that TSP50 deficiency leads to intestinal mucosal barrier damage (Li et al. 2024). Given the role of TSP50 in the regulation of intestinal homeostasis and development, we hypothesized that TSP50 might be involved in regulating the homeostasis of gut microbiota and in aging.

In this study, we generated NSCs‐specific TSP50 knockout mice and used 16S rRNA sequencing to evaluate the effect of TSP50 on gut microbiota, clarifying the physiological role of TSP50 in the regulation of aging by altering gut microbiota composition. We successfully alleviated aging‐related cognitive impairment and neuroinflammation caused by TSP50 deficiency through FMT and targeted intervention of TSP50. In summary, our data highlight the function of TSP50 and reveal mechanisms underlying the interaction between changes in gut microbiota composition and aging.

## 2 Materials and Methods

### 2.1 Mice

*TSP50* ^*flox/flox*^ (*TSP50* ^*fl/fl*^) mice, generated as previously described (Li et al. 2024), were used as controls in all experiments. Nestin‐Cre mice were purchased from GemPharmatech (Nanjing, China). *TSP50* ^*fl/fl*^ *Nestin* ^*Cre*^ mice were generated by crossing *TSP50* ^*fl/fl*^ mice with Nestin‐Cre mice. All mice were bred on a C57BL/6J background and kept in a specific pathogen‐free (SPF) environment. All animal protocols were approved by the Animal Advisory Committee of Northeast Normal University (NENU/IACUC, AP20191225). Mice aged between 18 and 24 months were considered old and used for behavioral tests.

### 2.2 Novel Object Recognition (NOR) Test

Experimental procedures were performed as described previously (Liu, Li, et al. 2021). On Day 1, mice were habituated to an arena without objects for 10 min. On Day 2, mice were habituated to the arena for 15 min with two identical objects. On Day 3, a familiar object was replaced with a novel one, and mice were again placed in the arena and allowed to explore for 10 min. Recognition index = (time spent exploring the novel objects − time spent exploring the familiar objects)/(time spent exploring the novel objects + time spent exploring the familiar objects).

### 2.3 Novel Object Location (NOL) Test

Experimental procedures were conducted as described previously (Wang et al. 2019). On Day 1, mice were habituated to an arena without objects for 10 min. On Day 2, mice were habituated to the arena for 15 min with two identical objects placed in two corners of the arena. On Day 3, mice were tested using the same two objects, one object placed in a familiar position, and the second object placed in a new novel corner of the arena. Mice were allowed to explore freely for 10 min. Recognition index = (time spent exploring a novel location ‐time spent exploring the familiar location)/ (time spent exploring a novel location + time spent exploring the familiar location).

### 2.4 Enzyme‐Linked Immunosorbent Assay (ELISA)

The removed mouse blood was allowed to stand at room temperature for 2 h, then centrifuged at 4000 rpm for 20 min, and the serum was carefully separated for testing. The hippocampal tissues were weighed and then added to pre‐cooled PBS (0.01 M, pH = 7.4) according to the weight/volume ratio of 1:9. The tissues were cut and homogenized on ice. The homogenate was subsequently centrifuged at 5000 *g* for 10 min, and the supernatant was taken for testing. Mouse adrenocorticotropic hormone (ACTH) ELISA kit, mouse corticotropin‐releasing hormone (CRH) ELISA kit, mouse corticosterone (CORT) ELISA kit, mouse tumor necrosis factor‐α (TNF‐α) ELISA kit, mouse interleukin‐6 (IL‐6) ELISA kit, and mouse lipopolysaccharide (LPS) ELISA kit (all from COIBO, Shanghai, China) were used to measure the levels of ACTH, CRH, CORT, TNF‐α, IL‐6, and LPS, respectively, according to the manufacturer's instructions.

### 2.5 Biochemical Assay

Serum samples stored at −80°C were used to measure biochemical indicators related to oxidative stress. Malondialdehyde (MDA) assay kit (TBA method), superoxide dismutase (SOD) assay kit (WST‐1 method), catalase (CAT) assay kit (visible light) and reduced glutathione (GSH) assay kit (all from Nanjing Jiancheng Bioengineering Institute, Nanjing, China) were used to measure the MDA levels, SOD activity, CAT activity, and GSH levels in serum of mice according to the manufacturer's instructions, respectively.

### 2.6 Immunofluorescence (IF) Staining

Mouse brain tissues were collected and placed in 4% paraformaldehyde solution, fixed for 6–18 h, dehydrated in 30% sucrose overnight, embedded in OCT, and samples were cut into 8 μm frozen sections. The frozen sections were first washed in PBS, blocked by 5% BSA in 0.2% Triton X‐100/PBS, and then incubated with specific primary antibodies overnight at 4°C: anti‐NESTIN (1:200; 19483‐1‐AP, Proteintech, Wuhan, China), anti‐TSP50 (1:100; ab181993, Abcam, Cambridge, United Kingdom). This was followed by incubation with the corresponding secondary antibodies: Cy3‐conjugated Goat Anti‐Rabbit IgG (H + L) (1:400; SA00009‐1, Proteintech) or FITC‐conjugated Goat Anti‐Mouse IgG (H + L) (1:400; SA00003‐1, Proteintech), and preserved in an anti‐fluorescence quenching solution containing DAPI. Images were acquired using a laser scanning confocal microscope (Zeiss, Oberkochen, Germany).

### 2.7 Senescence β‐Galactosidase (SA‐β‐Gal) Staining

Frozen sections of the brain tissues mentioned above were stained using a senescence β‐galactosidase (SA‐β‐Gal) staining kit (Beyotime, Shanghai, China) according to the manufacturer's instructions. The slides were mounted using glycerin gelatin and observed under an inverted microscope (Nikon, Tokyo, Japan).

### 2.8 Immunohistochemistry (IHC) Staining

Brain and colon tissues from mice were collected, fixed in 10% formalin solution, dehydrated in gradient ethanol, embedded in paraffin, and cut into 5 μm paraffin sections. IHC staining was performed as previously reported (Li et al. 2024). The paraffin sections were incubated with anti‐GFAP (16825‐1‐AP, Proteintech), anti‐IBA1 (26177‐1‐AP, Proteintech), and anti‐MUC2 (ab272692, Abcam) overnight at 4°C. The next day, the sections were incubated with streptavidin‐biotin complex and stained using DAB (ZSGB‐BIO, Beijing, China). Cell nuclei were counterstained with hematoxylin. Images were acquired via a Nikon Eclipse microscope (Nikon).

### 2.9 Nissl Staining

Paraffin sections of the brain tissues mentioned above were stained using a Nissl staining kit (Solarbio, Shanghai, China) according to the manufacturer's instructions. The slides were sealed with neutral gum and observed under a Nikon Eclipse microscope.

### 2.10 Hematoxylin and Eosin (HE) Staining

Paraffin sections of the colon tissues mentioned above were stained using an HE staining kit (Solarbio) according to the manufacturer's instructions. The slides were sealed with neutral gum and observed using a Nikon Eclipse microscope.

### 2.11 Alcian Blue Staining

Paraffin sections of colon tissue were stained with the Alcian blue staining kit (Solarbio) according to the manufacturer's instructions. The nuclei were labeled with a nuclear solid red staining solution. Images were acquired using a Nikon Eclipse microscope.

### 2.12 RNA Extraction and RT‐qPCR

Total RNA was isolated using Trizol reagent, and cDNAs were synthesized using a RT‐qPCR Kit (TransGen Biotech, Beijing, China) according to the manufacturer's instructions. Primers for RT‐qPCR detection were designed using Primer 6 software, and all primers were synthesized by Sangon Biotech (Shanghai, China). β‐ACTIN was used as a reference gene. The RT‐qPCR primers are listed as follows (5′‐3′): *TSP50*: CTCGGCAAGCCCAGACTAAC (forward) and CGAGAGTCCCCACCGTTTT (reverse); *TNF‐*α: CAGGCGGTGCCTATGTCTC (forward) and CGATCACCCCGAAGTTCAGTAG (reverse); *IL‐6*: CTGCAAGAGACTTCCATCCAG (forward) and AGTGGTATAGACAGGTCTGTTGG (reverse); *β‐ACTIN*: GGCTGTATTCCCCTCCATCG (forward) and CCAGTTGGTAACAATGCCATGT (reverse). The RT‐qPCR procedure was performed according to the instructions provided by the SYBR Green Kit (TransGen Biotech). The data were analyzed using the 2^−△△CT^ method.

### 2.13 Western Blot

The isolated animal tissues were weighed, and 600 μL RIPA lysis buffer was added to each 0.1 g sample, which was then fully homogenized. The cells were disrupted by ultrasonication at low temperature intervals for protein extraction. The extracted proteins were subjected to electrophoresis, transferred to a membrane, blocked, and incubated with primary antibodies: anti‐TSP50 (ab181993, Abcam), anti‐GFAP (16825‐1‐AP, Proteintech), anti‐IBA1 (26177‐1‐AP, Proteintech), anti‐iNOS (18985‐1‐AP, Proteintech), anti‐Occludin (27260‐1‐AP, Proteintech), anti‐Claudin1 (28674‐1‐AP, Proteintech), anti‐β‐Actin (66009‐1‐Ig, Proteintech) and corresponding secondary antibodies (Proteintech) according to the manufacturer's instructions. The immunoblots were visualized using an enhanced chemiluminescence (ECL) solution (Beyotime, Beijing, China). Protein bands were quantified using ImageJ software.

### 2.14 AAV‐PHP.eB Vectors Delivery

Empty AAV‐PHP.eB vectors and TSP50‐overexpressing AAV‐PHP.eB vectors were constructed by Vigene Biosciences (Shandong, China). The empty and TSP50‐overexpressing AAV‐PHP.eB vectors were injected into 18‐month‐old *TSP50* ^*fl/fl*^ *Nestin* ^*Cre*^ mice via the tail vein at a dose of 5 × 10^13^ genome copies per mouse.

### 2.15 Bacterial Translocation Assay

The mesentery, liver, spleen, and kidney of mice were removed under sterile conditions, 9 mL of saline was added to each 1 g sample, which was then fully homogenized, and 100 μL of the homogenate supernatant was incubated on Luria‐Bertani (LB) agar for 24 h at 37°C. Any bacteria growing on the plates was considered positive.

### 2.16 Antibiotic Treatment

To deplete the gut microbiota, aged mice were treated with a cocktail of antibiotics in autoclaved drinking water for 4 weeks at the following concentrations: 1 g/L ampicillin, 1 g/L metronidazole, 1 g/L neomycin, and 0.5 g/L vancomycin (all from Solarbio). Fresh antibiotic‐containing water was provided every 48 h to ensure stability. This administration procedure was performed in accordance with previous studies aiming to alter the gut microbiome composition (Jiang et al. 2019).

### 2.17 Fecal Microbiota Transplantation (FMT)

To better colonize the gut microbiota, recipient mice were treated with a 1‐week antibiotic cocktail. After that, fresh feces from donor mice were collected daily during FMT, and 1 mL of PBS was added to each 50 mg sample, which was vortexed thoroughly for 3 min and allowed to precipitate naturally for 5 min to collect the supernatant. Each recipient mouse received 200 μL of the supernatant daily by gavage for 8 weeks.

### 2.18 16S rRNA Sequencing

Fecal microbiota DNA was extracted using MagPure Stool DNA KF Kit B (Magen Biotechnology, Beijing, China). Qualified genomic DNA samples were used for PCR amplification, and the PCR products were purified using Agencourt AMPure XP magnetic beads. The Agilent 2100 Bioanalyzer was used to detect the fragment size range and concentration of the libraries, and the qualified libraries were sequenced according to the insert size. Raw sequencing data were processed to obtain clean data. FLASH software was used for sequence splicing, and the overlapping regions were used to assemble the paired‐end reads into a single sequence, thereby obtaining tags in the hypervariable region. The USEARCH software was used to cluster the stitched tags into OUTs. OTU sequences were aligned with the Greengenes database using the RDP classifier software for species annotation. Based on the OTU and annotation results, species complexity analysis, species difference analysis, and association analysis were performed. These protocols were conducted by the Beijing Genomics Institution (Shenzhen, China).

### 2.19 Short‐Chain Fatty Acids (SCFAs) Assay

The fecal samples were thawed at 4°C, and 100 mg of each sample was mixed with 50 μL of 20% phosphoric acid in water and 500 μL of isopropyl ether containing 500 μM 4‐methylvaleric acid and vortexed thoroughly. The mixture was centrifuged at 14,000 *g* for 20 min at 4°C, and the supernatants were collected for gas chromatography–mass spectrometry (GC–MS) analysis. Samples were separated using an Agilent DB‐FFAP capillary column gas chromatography system. Mass spectrometry was performed on an Agilent 5977B MSD mass spectrometer. The MSD ChemStation software was used to extract chromatographic peak areas and retention times. The content of SCFAs was calculated by constructing a standard curve. The protocols were conducted by Applied Protein Technology (Shanghai, China).

### 2.20 Statistical Analysis

GraphPad Prism 9.0 and SPSS software were used for statistical analysis. All data from at least three independent replicates were presented as mean ± SEM. Two‐tailed unpaired Student's *t*‐tests, one‐way ANOVA, and paired sample Wilcoxon signed‐rank tests were used for data analysis. The statistical significance was as follows: ns means no significance, *p < 0.05, **p < 0.01, ***p < 0.001, ****p < 0.0001.

## 3 Results

### 3.1 TSP50 Expression Is Decreased in the Hippocampus of Both the Aged Individuals and Mice

**FIGURE 1:**
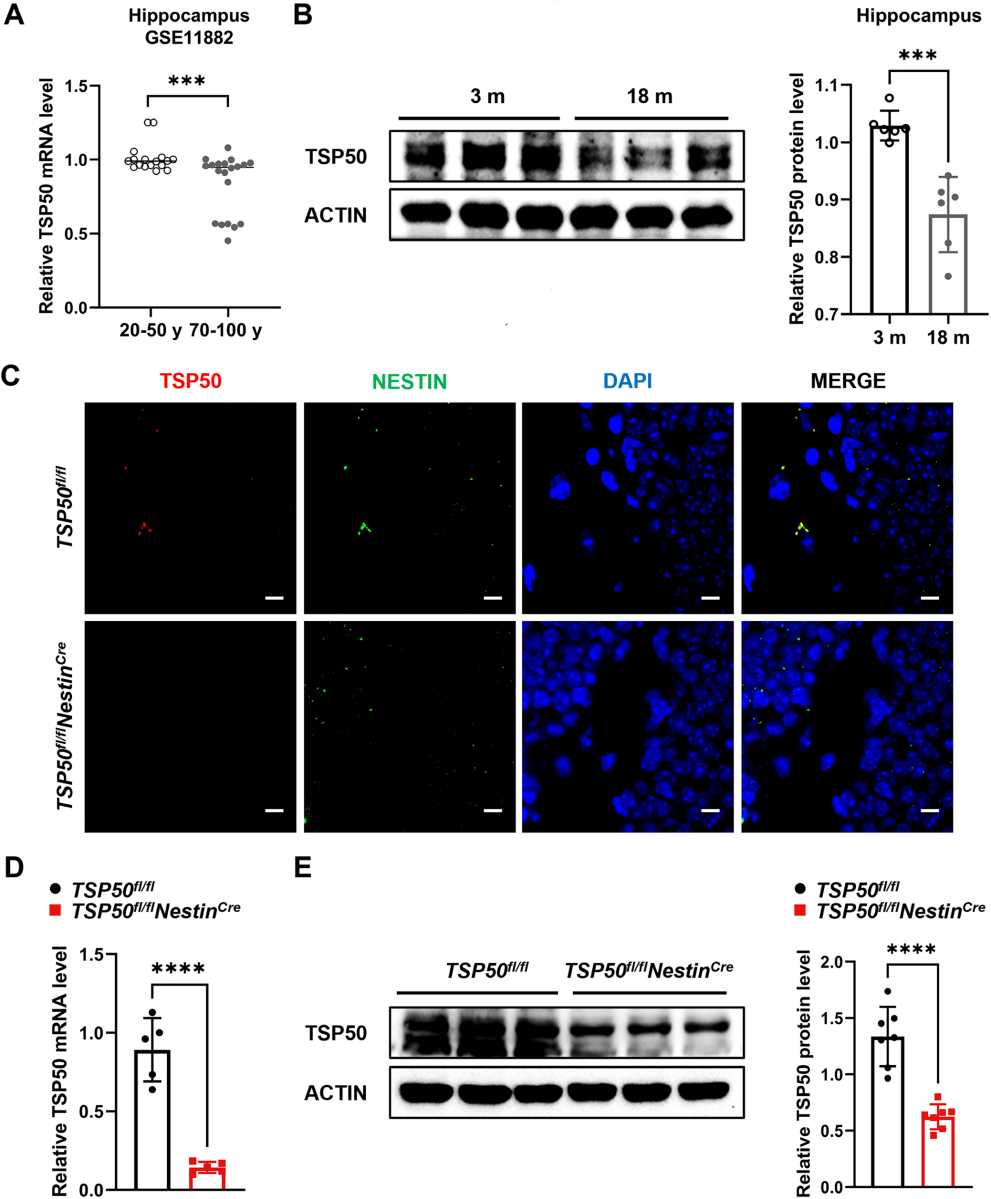
TSP50 expression decreases during aging. (A) TSP50 expression is downregulated in the hippocampus of the aging population according to the NCBI GEO database (GSE11882: People aged 20–50 years, *n* = 17, people aged 70–100 years, *n* = 20). (B) Western blot analysis and quantification of TSP50 protein levels in the hippocampus of aging mice. (C) Representative images of IF staining of TSP50 and NESTIN expression in the hippocampus of mice. Scale bar: 10 μm. (D) RT‐qPCR analysis of TSP50 mRNA levels in the hippocampus of mice. (E) Western blot analysis and quantification of TSP50 protein levels in the hippocampus of mice. Values are expressed as means ± SEM, ***p < 0.001, ****p < 0.0001.

To explore the potential role of TSP50 in aging, we analyzed TSP50 transcription levels in the hippocampus of aging populations using the GEO dataset. By analyzing the data from the GSE11882 dataset, we found that the TSP50 transcription levels in the hippocampus of aging people over 70 years old were significantly lower than those of younger individuals between 20 and 50 years old (Figure 1A). Consistent with this finding, TSP50 expression was also significantly reduced in the hippocampus of 18‐month‐old aged mice compared with 2‐month‐old young mice (Figure 1B). These results suggest that TSP50 in NSCs may play an important role in aging.

### 3.2 TSP50 Deficiency in NSCs Accelerates Aging in Mice

To further investigate the role of TSP50 in aging, we generated NSC‐specific TSP50 knockout mice: *TSP50* ^*fl/fl*^ *Nestin* ^*Cre*^ mice. IF staining showed that TSP50 was absent from NSCs in the hippocampus of *TSP50* ^*fl/fl*^ *Nestin* ^*Cre*^ mice (Figure 1C). The results of RT‐qPCR and Western blot experiments further confirmed that hippocampal TSP50 levels were significantly reduced in *TSP50* ^*fl/fl*^ *Nestin* ^*Cre*^ mice (Figure 1D,E), indicating the efficacy of the deletion.

**FIGURE 2:**
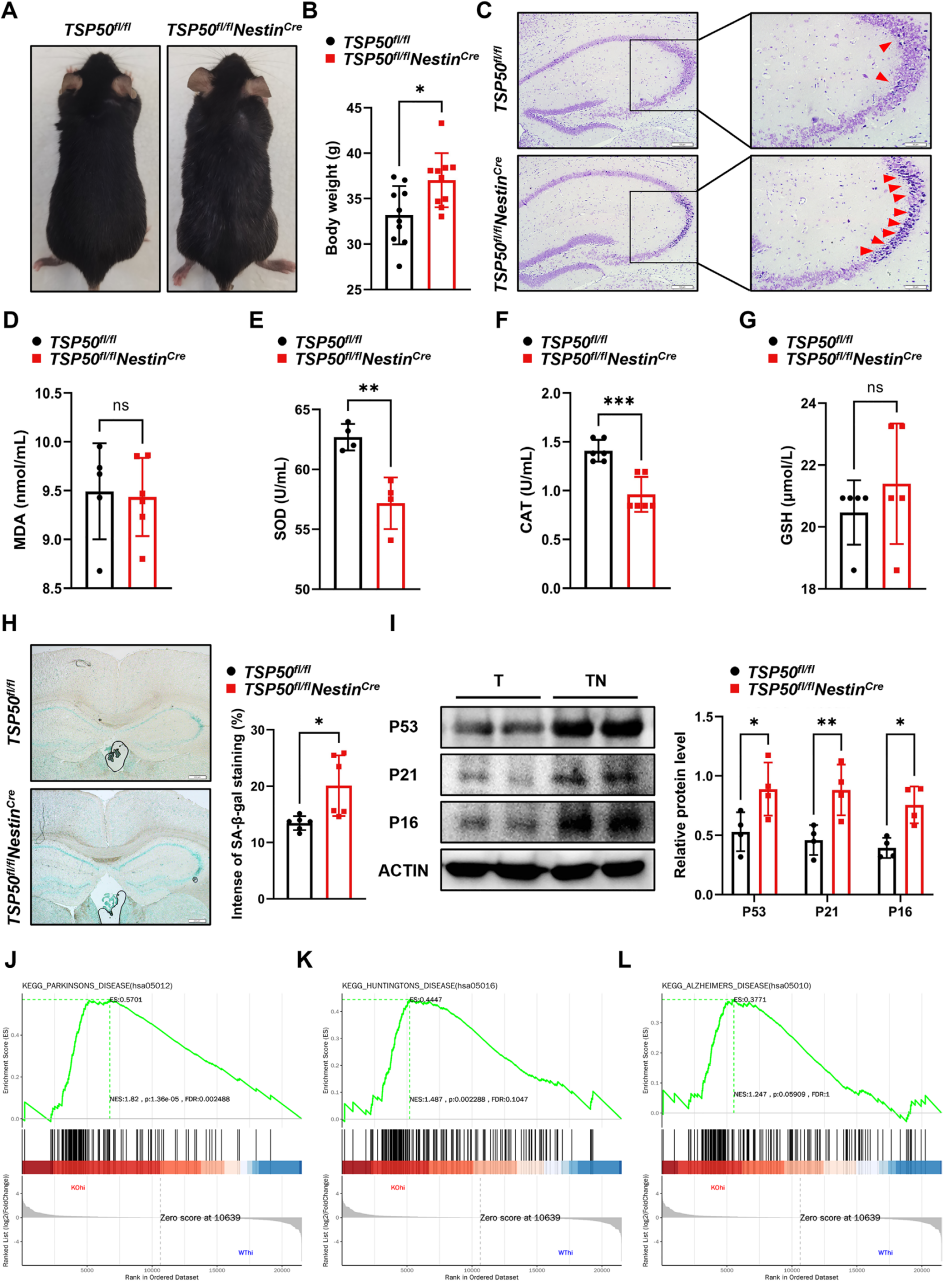
TSP50 deficiency in NSCs accelerates mouse aging. (A) Representative images of 18‐month‐old control and *TSP50* ^*fl/fl*^ *Nestin* ^*Cre*^ mice. (B) Body weights of aged mice. (C) Representative Nissl staining images of the hippocampus of aged mice. Scale bar: Left images 100 μm, right images 50 μm. (D) Serum MDA levels in aged mice. (E) Serum SOD activity in aged mice. (F) Serum CAT activity in aged mice. (G) Serum GSH levels in aged mice. (H) Representative SA‐β‐Gal staining images of the hippocampus of aged mice and quantitative analysis. Scale bar: 200 μm. (I) Western blot analysis and quantification of P53, P21, and P16 protein levels in the hippocampus of aged mice. (T: *TSP50* ^*fl/fl*^; TN: *TSP50* ^*fl/fl*^ *Nestin* ^*Cre*^) (J) GSEA of the PD signaling pathway. (K) GSEA of the HD signaling pathway. (L) GSEA of the AD signaling pathway. Values are expressed as means ± SEM, ns (no significance), *p < 0.05, **p < 0.01, ***p < 0.001.

Furthermore, the aged *TSP50* ^*fl/fl*^ *Nestin* ^*Cre*^ mice exhibited a more pronounced senescent phenotype, including more distressed and duller fur, a significant increase in the number of white hairs, and a significant increase in body weight (Figure 2A,B). Nissl staining results showed that hippocampal neurons in *TSP50* ^*fl/fl*^ *Nestin* ^*Cre*^ mice were disarranged and accompanied by more cell necrosis than those in control mice (Figure 2C). Oxidative stress is an important factor in cellular senescence (Barbouti et al. 2020). Therefore, we further examined the MDA levels, SOD activity, CAT activity, and GSH levels in the serum of aging mice. The results showed that compared with the control mice, *TSP50* ^*fl/fl*^ *Nestin* ^*Cre*^ mice exhibited no significant changes in MDA and GSH levels, but had significantly decreased SOD activity and CAT activity (Figure 2D–G). These results indicate that TSP50 deficiency impairs antioxidant capacity and enhances the oxidative stress response in aging mice. SA‐β‐Gal is an important biomarker of senescence that gradually accumulates during cellular senescence. We found that SA‐β‐Gal levels were increased in the hippocampus of *TSP50* ^*fl/fl*^ *Nestin* ^*Cre*^ mice (Figure 2H). In addition, the expression of senescence‐related proteins including P53, P21, and P16 was also significantly increased in the hippocampus of *TSP50* ^*fl/fl*^ *Nestin* ^*Cre*^ mice (Figure 2I), indicating that TSP50 deficiency in NSCs accelerates brain aging in mice.

To gain insight into the affected biological processes of TSP50 expression in aging, gene set enrichment analysis (GSEA) was performed on transcriptome sequencing data from the mouse hippocampus. The results showed that TSP50 deletion activated aging‐related neurodegenerative disease processes, such as those seen in PD, HD, and AD (Figure 2J–L). Together, these data show that TSP50 deficiency in NSCs enhances the oxidative stress response and accelerates aging in mice.

### 3.3 TSP50 Deficiency in NSCs Aggravates Cognitive Impairment and Neuroinflammation in Aging Mice

**FIGURE 3:**
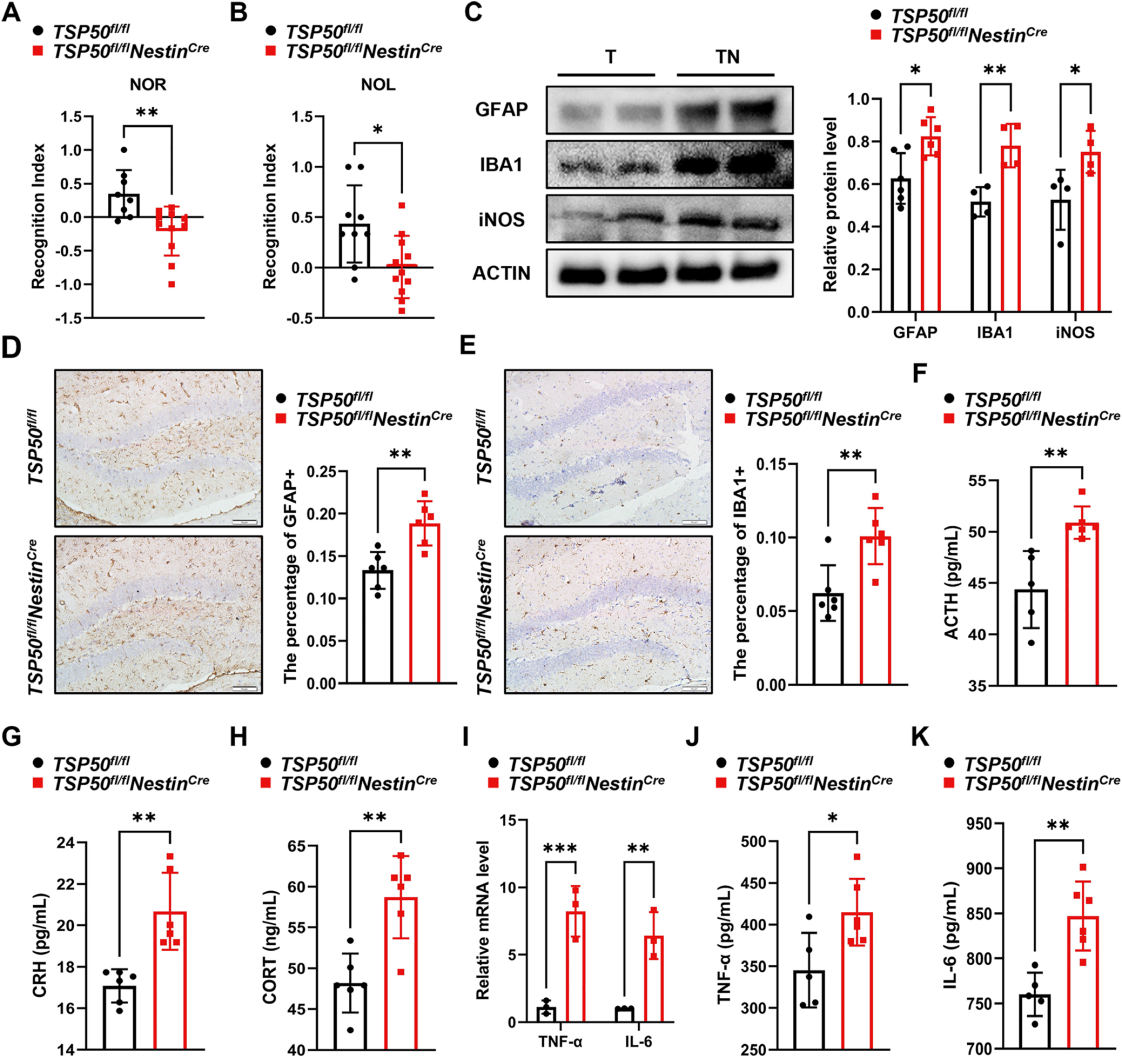
TSP50 deficiency in NSCs aggravates cognitive impairment and neuroinflammation in aging mice. (A) NOR test results of aged mice. (B) NOL test results of aged mice. (C) Western blot analysis and quantification of GFAP, IBA1, and iNOS protein levels in the hippocampus of aged mice. (T: *TSP50* ^*fl/fl*^; TN: *TSP50* ^*fl/fl*^ *Nestin* ^*Cre*^) (D) Representative images of IHC staining for GFAP expression in the hippocampus of aged mice, along with quantitative analysis. Scale bar: 50 μm. (E) Representative images of IHC staining for IBA1 expression in the hippocampus of aged mice, along with quantitative analysis. Scale bar: 50 μm. (F) ELISA analysis of serum ACTH levels in aged mice. (G) ELISA analysis of serum CRH levels in aged mice. (H) ELISA analysis of serum CORT levels in aged mice. (I) RT‐qPCR analysis of hippocampal TNF‐α and IL‐6 mRNA levels in aged mice. (J) ELISA analysis of hippocampal TNF‐α levels in aged mice. (K) ELISA analysis of hippocampal IL‐6 levels in aged mice. Values are expressed as means ± SEM, *p < 0.05, **p < 0.01.

Cognitive dysfunction is a universal feature of aging and neurodegenerative diseases (Gonzales et al. 2022). To investigate the effect of TSP50 deletion on cognitive function during aging, we performed NOR and NOL experiments in aging mice. The results indicated that aged *TSP50* ^*fl/fl*^ *Nestin* ^*Cre*^ mice had decreased cognitive ability (Figure 3A,B). Studies have shown that neuroinflammation is one of the main causes of cognitive decline and is an early lesion of neurodegenerative diseases (Zhang et al. 2023), with microglia and astrocytes being major players (Singh 2022). We observed that the number of astrocytes and microglia in the hippocampus of aged *TSP50* ^*fl/fl*^ *Nestin* ^*Cre*^ mice was significantly increased (Figure 3C–E). In addition, the level of the neuroinflammatory mediator iNOS was also significantly increased in the hippocampus of aged *TSP50* ^*fl/fl*^ *Nestin* ^*Cre*^ mice (Figure 3C), indicating that the deficiency of TSP50 in NSCs aggravated neuroinflammation in aged mice. The activation of the hypothalamic–pituitary–adrenal (HPA) axis can affect the expression of inflammatory factors and the activation of microglia, which plays an important role in the regulation of the central inflammatory response (Woodburn et al. 2021). The ELISA results showed that serum ACTH, CRH, and CORT levels were all significantly increased in aged *TSP50* ^*fl/fl*^ *Nestin* ^*Cre*^ mice, suggesting that TSP50 deletion causes abnormal HPA axis activation in aged mice (Figure 3F–H). Furthermore, we observed significant increases in both mRNA and protein levels of TNF‐α and IL‐6, two key neuroinflammatory markers, in the hippocampus of aged *TSP50* ^*fl/fl*^ *Nestin* ^*Cre*^ mice compared with those of aged control mice (Figure 3I–K). In conclusion, these data suggest that TSP50 deficiency in NSCs aggravates cognitive impairment and neuroinflammation in aging mice.

### 3.4 TSP50 Deficiency in NSCs Promotes Intestinal Barrier Damage and Systemic Inflammation in Aging Mice

Recent studies have shown that there is a close link between the damage of the intestinal mucosal barrier and neuroinflammation (Agirman et al. 2021). The disruption of the intestinal barrier may lead to the entry of gut‐derived molecular and microbial pathogens into the central nervous system, triggering or exacerbating neuroinflammation (Solanki et al. 2023). To explore whether the increased neuroinflammation in aged *TSP50* ^*fl/fl*^ *Nestin* ^*Cre*^ mice was associated with the intestinal mucosal barrier, we examined the intestinal mucosal barrier in these aged mice. HE staining showed that the colonic crypts of aged *TSP50* ^*fl/fl*^ *Nestin* ^*Cre*^ mice were disorganized, the depth of the crypts was decreased, and inflammatory cell infiltration was increased (Figure S1A). Additionally, we found that tight junction protein expression, goblet cell number, and mucin secretion were significantly reduced in the colon of aged *TSP50* ^*fl/fl*^ *Nestin* ^*Cre*^ mice (Figure S1B–D), indicating that TSP50 deficiency in NSCs contributes to the impairment of the intestinal mucosal barrier in aged mice.

Intestinal mucosal barrier damage can lead to increased barrier permeability, thereby promoting systemic inflammation (Di Vincenzo et al. 2024). Increased levels of LPS in serum are considered to be a marker of increased intestinal mucosal permeability (Candelli et al. 2021). We found that aged *TSP50* ^*fl/fl*^ *Nestin* ^*Cre*^ mice had significantly higher serum LPS levels compared with aged control mice (Figure S1E). The bacterial translocation assay revealed that *TSP50* ^*fl/fl*^ *Nestin* ^*Cre*^ mice exhibited bacterial translocation in the mesentery (Figure S1F), indicating increased intestinal mucosal permeability and direct contact between gut microbiota and intestinal epithelium in aged *TSP50* ^*fl/fl*^ *Nestin* ^*Cre*^ mice. Moreover, we observed that the levels of serum proinflammatory cytokines TNF‐α and IL‐6 were significantly elevated in aged *TSP50* ^*fl/fl*^ *Nestin* ^*Cre*^ mice (Figure S1G,H). These results collectively suggest that TSP50 deficiency in NSCs leads to intestinal mucosal barrier damage in aged mice, resulting in the leakage of intestinal molecules and triggering a series of inflammatory responses.

### 3.5 TSP50 Deficiency in NSCs Alters the Intestinal Microbiota and Reduces SCFAs Production in Aging Mice

**FIGURE 4:**
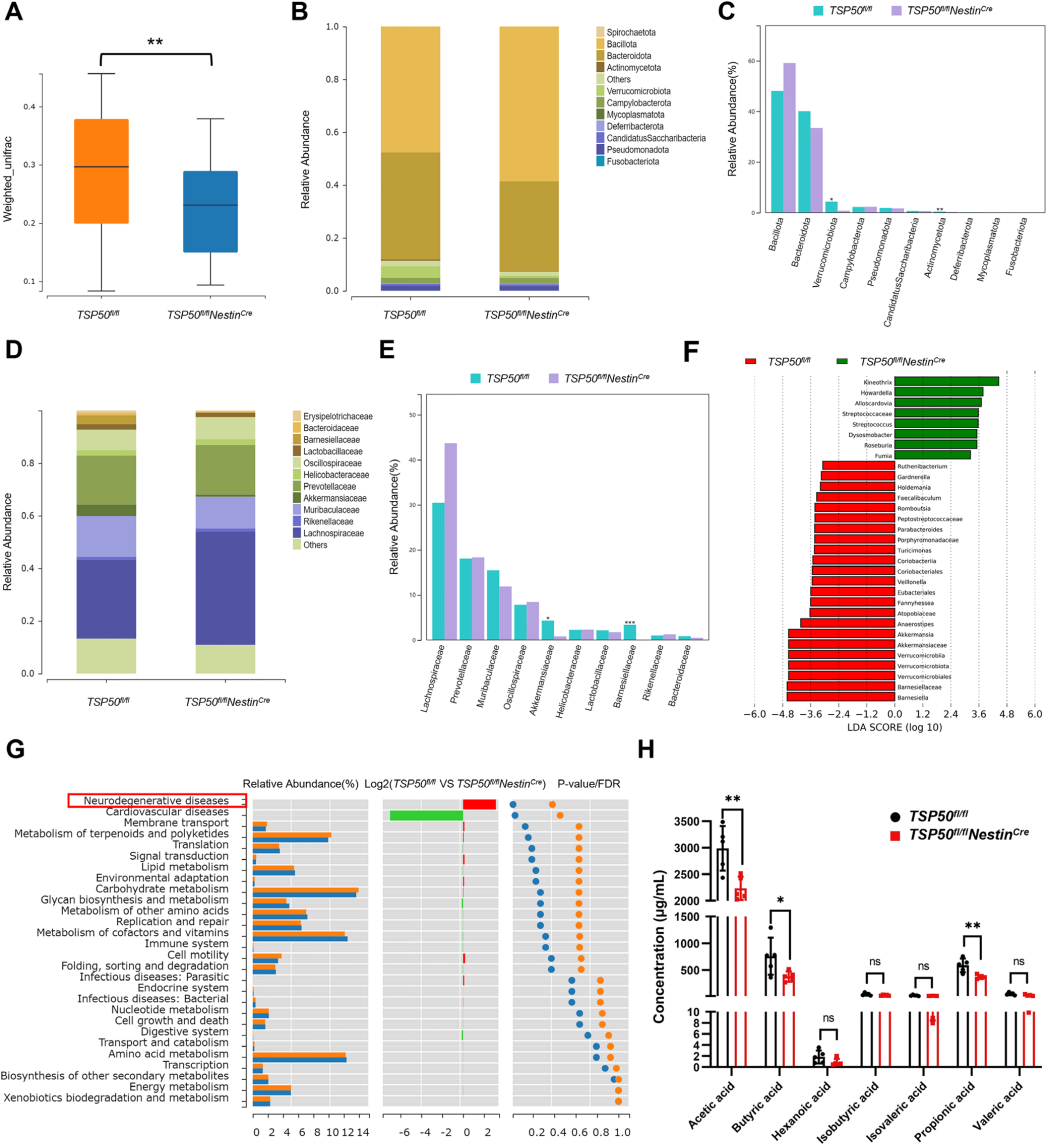
TSP50 deficiency in NSCs alters the intestinal microbiota and reduces SCFAs production in aging mice. (A) PCoA analysis of fecal microbiota from aged mice using weighted UniFrac distances. (B) Histogram of phylum‐level species composition. (C) Histogram of species difference comparison for the top 10 abundances at phylum level. (D) Histogram of family‐level species composition. (E) Histogram of species difference comparison for the top 10 abundances at the family level. (F) LEfSe analysis. (G) Functional difference analysis of the gut microbiota in aged mice. (H) GC–MS analysis of fecal SCFA levels in aged mice. Values are expressed as means ± SEM, ns (no significance), *p < 0.05, **p < 0.01, ***p < 0.001.

A growing number of studies have shown that alterations in gut microbiota can lead to increased permeability of the intestinal barrier and blood–brain barrier. These changes in permeability may facilitate the accumulation of molecules (such as LPS) and metabolites (such as SCFAs) derived from gut microbes in the brain, promoting the subsequent transition of the brain environment to a proinflammatory state, thereby creating conditions for the development of neurodegenerative diseases, such as PD, HD, and AD (Yadav et al. 2023). To explore whether the cognitive impairment and neuroinflammation caused by TSP50 deletion are associated with the gut microbiota, the gut microbiota of aged control mice and aged *TSP50* ^*fl/fl*^ *Nestin* ^*Cre*^ mice were examined using 16S rRNA sequencing. Principal coordinate analysis (PCoA) based on weighted UniFrac distance revealed significant differences in gut microbiota composition and structure between aged control and aged *TSP50* ^*fl/fl*^ *Nestin* ^*Cre*^ mice (Figure 4A). Species composition analysis revealed that in aged *TSP50* ^*fl/fl*^ *Nestin* ^*Cre*^ mice, the abundance of Verrucomicrobia and Actinobacteria at the phylum level is lower, and the abundance of *Akkermansiaceae* and *Barnesiellaceae* at the family level is also lower (Figure 4B–E). Linear discriminant analysis effect size (LEfSe) showed that *Barnesiella* and *Akkermansia* were the dominant bacteria in aged control mice, while *Kineothrix* and *Howardella* were the dominant bacteria in aged *TSP50* ^*fl/fl*^ *Nestin* ^*Cre*^ mice (Figure 4F). Furthermore, functional difference analysis of gut microbiota showed that pathways related to neurodegenerative diseases were significantly upregulated in aged *TSP50* ^*fl/fl*^ *Nestin* ^*Cre*^ mice compared with aged control mice (Figure 4G). SCFAs are beneficial metabolites of gut microbiota metabolism, and their anti‐inflammatory effects have been widely reported (Xiong et al. 2022). Determination of SCFAs concentrations in fecal samples showed that acetate, butyrate, and propionic acid levels were significantly reduced in aged *TSP50* ^*fl/fl*^ *Nestin* ^*Cre*^ mice (Figure 4H). Taken together, these results suggest that TSP50 may affect neuroinflammation and cognitive function in aged mice by regulating gut microbiota composition as well as SCFAs production.

### 3.6 Exacerbation of Cognitive Impairment and Neuroinflammation in Aged *TSP50* ^*fl*^ ^*/*^ ^*fl*^ *Nestin* ^*Cre*^ Mice Depends on the Gut Microbiota

To determine whether microbiota‐dependent mechanisms contribute to aggravated age‐related cognitive impairment and neuroinflammation, aged control and *TSP50* ^*fl/fl*^ *Nestin* ^*Cre*^ mice were treated with an antibiotic cocktail (ABX) for 4 weeks to deplete their gut microbiota. Following antibiotic treatment, we observed that aged *TSP50* ^*fl/fl*^ *Nestin* ^*Cre*^ mice and control mice exhibited comparable cognitive performance in NOR and NOL tests (Figure S2A,B). Furthermore, Western blot analysis revealed similar levels of GFAP, IBA1, and iNOS in the hippocampus of both groups (Figure S2C), and ELISA showed no significant differences in serum or hippocampal TNF‐α and IL‐6 levels (Figure S2D–G). These results collectively demonstrate that the exacerbation of neuroinflammation and cognitive decline in aged NSCs‐specific TSP50 knockout mice relies on the presence of gut microbiota, as the phenotypes were abolished upon microbiota depletion.

### 3.7 FMT Alleviates Cognitive Impairment and Neuroinflammation in Aged *TSP50* ^*fl*^ ^*/*^ ^*fl*^ *Nestin* ^*Cre*^ Mice

To further confirm the role of gut microbiota in TSP50 knockout‐induced cognitive function and neuroinflammation in aged mice, we performed FMT experiments in which fecal microbiota from aged control mice were transplanted into aged *TSP50* ^*fl/fl*^ *Nestin* ^*Cre*^ mice and fecal microbiota from aged *TSP50* ^*fl/fl*^ *Nestin* ^*Cre*^ mice were transplanted into aged control mice. We found that aged *TSP50* ^*fl/fl*^ *Nestin* ^*Cre*^ mice transplanted with feces from aged control mice exhibited improved cognitive performance, whereas aged control mice transplanted with feces from aged *TSP50* ^*fl/fl*^ *Nestin* ^*Cre*^ mice showed decreased cognitive performance (Figure S3A,B). Consistent with this finding, the number of astrocytes and microglia, iNOS content, and HPA axis activity in the hippocampus of aged *TSP50* ^*fl/fl*^ *Nestin* ^*Cre*^ mice were significantly reduced after FMT treatment and largely returned to normal levels (Figure S3C–F). These results indicate that FMT can alleviate cognitive dysfunction and neuroinflammation in aged *TSP50* ^*fl/fl*^ *Nestin* ^*Cre*^ mice. Notably, the levels of inflammatory factors in the serum of aged *TSP50* ^*fl/fl*^ *Nestin* ^*Cre*^ mice after FMT treatment were similar to those of aged control mice, while the levels of inflammatory factors in the serum of aged control mice after FMT treatment were significantly increased (Figure S3G,H), suggesting that gut microbiota may play a key role in the occurrence and development of neuroinflammation.

### 3.8 TSP50 Supplementation Rescues Cognitive Impairment and Neuroinflammation in Aged *TSP50* ^*fl*^ ^*/*^ ^*fl*^ *Nestin* ^*Cre*^ Mice

**FIGURE 5:**
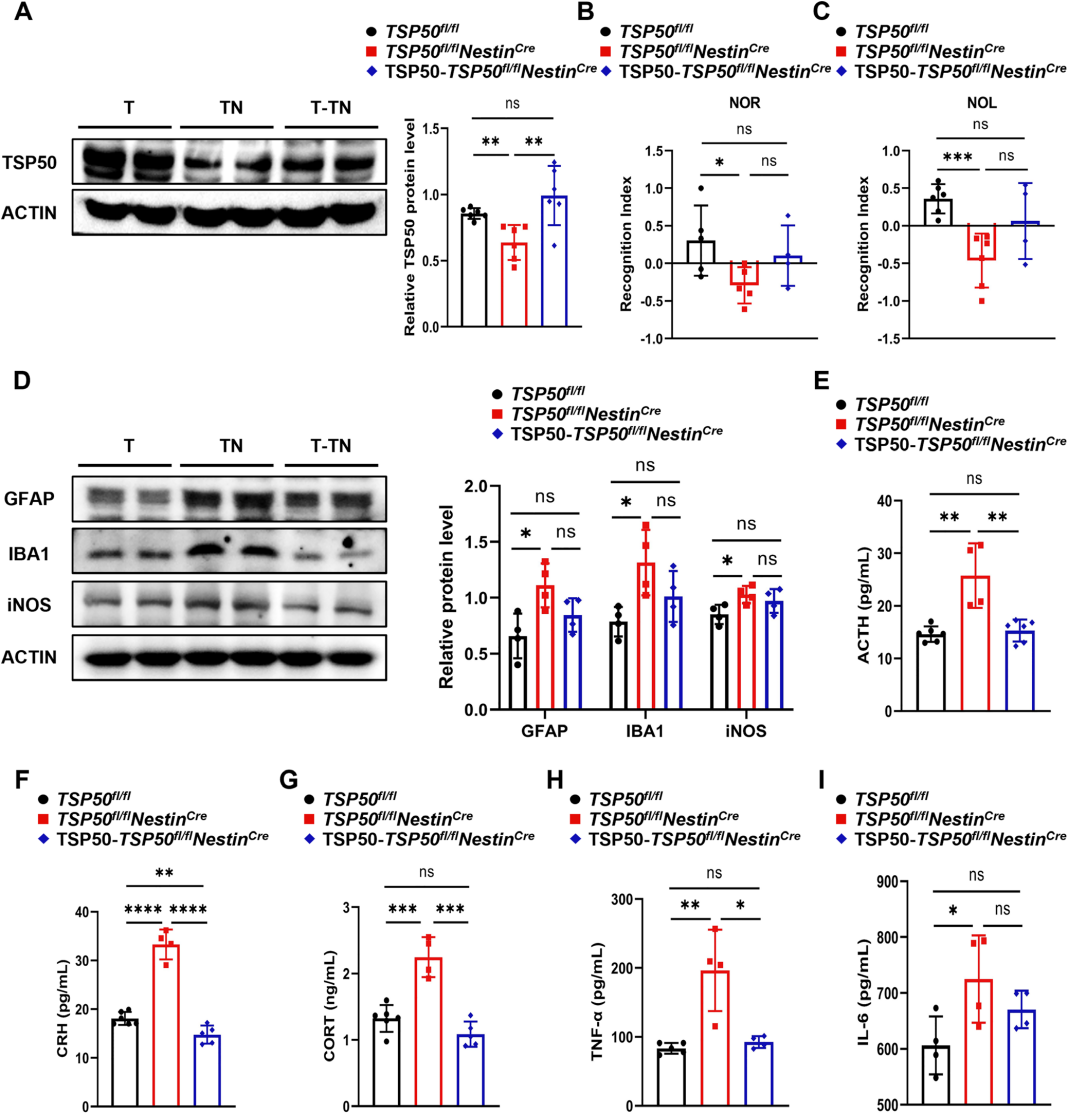
TSP50 supplementation rescues cognitive impairment and neuroinflammation in aged *TSP50* ^*fl/fl*^ *Nestin* ^*Cre*^ mice. (A) Western blot analysis and quantification of TSP50 protein levels in the hippocampus of aged mice after TSP50 supplementation. (T: *TSP50* ^*fl/fl*^; TN: *TSP50* ^*fl/fl*^ *Nestin* ^*Cre*^; T‐TN: TSP50‐*TSP50* ^*fl/fl*^ *Nestin* ^*Cre*^) (B) NOR test results of aged mice after TSP50 supplementation. (C) NOL test results of aged mice after TSP50 supplementation. (D) Western blot analysis and quantification of GFAP, IBA1, and iNOS protein levels in the hippocampus of aged mice after TSP50 supplementation. (T: *TSP50* ^*fl/fl*^; TN: *TSP50* ^*fl/fl*^ *Nestin* ^*Cre*^; T‐TN: TSP50‐*TSP50* ^*fl/fl*^ *Nestin* ^*Cre*^) (E) ELISA analysis of serum ACTH levels in aged mice after TSP50 supplementation. (F) ELISA analysis of serum CRH levels in aged mice after TSP50 supplementation. (G) ELISA analysis of serum CORT levels in aged mice after TSP50 supplementation. (H) ELISA analysis of serum TNF‐α levels in aged mice after TSP50 supplementation. (I) ELISA analysis of serum IL‐6 levels in aged mice after TSP50 supplementation. Values are expressed as means ± SEM, ns (no significance), *p < 0.05, **p < 0.01, ***p < 0.001, ****p < 0.0001.

To further determine the role of TSP50 in regulating cognitive function and neuroinflammation in aging mice, TSP50 overexpressing AAV‐PHP.eB vectors were administered to aged *TSP50* ^*fl/fl*^ *Nestin* ^*Cre*^ mice via tail vein injection for TSP50 replenishment. Six weeks after injection, the protein expression levels of TSP50 in the hippocampus of aged *TSP50* ^*fl/fl*^ *Nestin* ^*Cre*^ mice were significantly increased (Figure 5A), confirming the effectiveness of TSP50 restoration. Next, we tested the cognitive function and neuroinflammation in aged *TSP50* ^*fl/fl*^ *Nestin* ^*Cre*^ mice after TSP50 supplementation. The NOL and NOR results suggested that although the cognitive ability of aged *TSP50* ^*fl/fl*^ *Nestin* ^*Cre*^ mice did not significantly improve after TSP50 supplementation, it essentially returned to normal (Figure 5B,C). Consistently, the number of astrocytes and microglia, as well as the levels of iNOS in the hippocampus of aged *TSP50* ^*fl/fl*^ *Nestin* ^*Cre*^ mice, were also restored to similar levels as those of aged control mice after TSP50 supplementation (Figure 5D). In addition, we observed that overexpression of TSP50 significantly suppressed HPA axis activation and the expression of pro‐inflammatory cytokines TNF‐α and IL‐6 in the serum of aged *TSP50* ^*fl/fl*^ *Nestin* ^*Cre*^ mice (Figure 5E–I). Collectively, the above results consistently demonstrate that TSP50 supplementation can improve cognitive impairment and neuroinflammation in aged *TSP50* ^*fl/fl*^ *Nestin* ^*Cre*^ mice.

### 3.9 TSP50 Replenishment Restores Gut Microbiota and SCFAs Levels in Aged *TSP50* ^*fl*^ ^*/*^ ^*fl*^ *Nestin* ^*Cre*^ Mice

**FIGURE 6:**
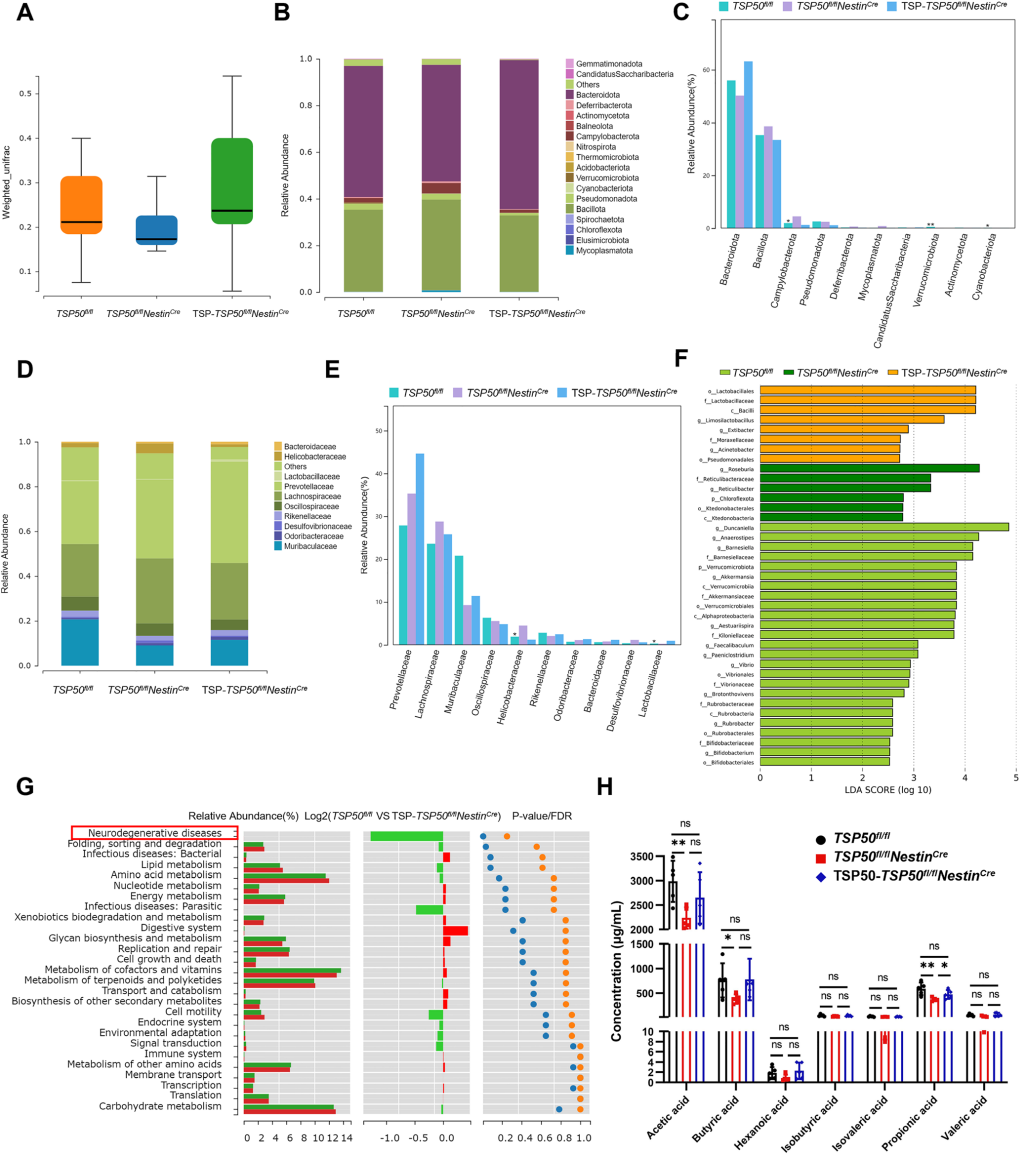
TSP50 replenishment restores gut microbiota and SCFAs levels in aged *TSP50* ^*fl/fl*^ *Nestin* ^*Cre*^ mice. (A) PCoA analysis of fecal microbiota from aged mice using weighted UniFrac distances after TSP50 replenishment. (B) Histogram of phylum‐level species composition after TSP50 replenishment. (C) Histogram comparing the top 10 phylum‐level species differences after TSP50 replenishment. (D) Histogram of family‐level species composition after TSP50 replenishment. (E) Histogram comparing the top 10 family‐level species differences after TSP50 replenishment. (F) LEfSe analysis. (G) Functional difference analysis of gut microbiota in aged mice after TSP50 replenishment. (H) GC–MS analysis of fecal SCFA levels in aged mice after TSP50 replenishment. Values are expressed as means ± SEM, ns (no significance), *p < 0.05, **p < 0.01.

To further confirm that TSP50 suppresses cognitive impairment and neuroinflammation in aging mice by regulating gut microbiota, we performed 16S rRNA sequencing of fecal microbiota in aged *TSP50* ^*fl/fl*^ *Nestin* ^*Cre*^ mice after TSP50 supplementation. PCoA analysis showed that the gut microbiota diversity of aged *TSP50* ^*fl/fl*^ *Nestin* ^*Cre*^ mice was significantly different from that of aged control mice, whereas the gut microbiota diversity of aged *TSP50* ^*fl/fl*^ *Nestin* ^*Cre*^ mice after TSP50 supplementation was similar to that of aged control mice (Figure 6A). Species composition analysis also showed that the composition and structure of gut microbiota in aged *TSP50* ^*fl/fl*^ *Nestin* ^*Cre*^ mice were significantly different from those in aged control mice, while the composition and structure of gut microbiota in aged *TSP50* ^*fl/fl*^ *Nestin* ^*Cre*^ mice after TSP50 supplementation were similar to those in aged control mice. Specifically, in aged *TSP50* ^*fl/fl*^ *Nestin* ^*Cre*^ mice, at the phylum level, the abundance of Verrucomicrobia and Cyanobacteriota is lower, while that of Campylobacterota is higher. At the family level, the abundance of *Lactobacillaceae* is lower, while that of *Helicobacteraceae* is higher. After TSP50 supplementation, the relative abundance of these bacteria returned to normal (Figure 6B–E). LEfSe analysis showed that the dominant bacterial species in the gut of aged *TSP50* ^*fl/fl*^ *Nestin* ^*Cre*^ mice changed to the beneficial bacterium *Lactobacillus* after TSP50 supplementation (Figure 6F), indicating that TSP50 indeed plays a crucial role in regulating gut microbiota. Furthermore, functional analysis of gut microbiota showed that pathways associated with neurodegenerative diseases were significantly downregulated in aged *TSP50* ^*fl/fl*^ *Nestin* ^*Cre*^ mice after TSP50 supplementation compared with aged control mice (Figure 6G). The results of SCFAs assay showed that the content of SCFAs in aged *TSP50* ^*fl/fl*^ *Nestin* ^*Cre*^ mice was also restored to normal after TSP50 supplementation (Figure 6H). In summary, these results strongly suggest that TSP50 can affect cognitive function and neuroinflammation in aged mice by regulating the composition of gut microbiota and the production of SCFAs.

## 4 Discussion

In this study, we found that the expression of TSP50 significantly decreased with age in both human and mouse models. The knockout of TSP50 in NSCs of mice led to accelerated aging, aggravated aging‐related cognitive impairment, and neuroinflammation in mice. In addition, aged *TSP50* ^*fl/fl*^ *Nestin* ^*Cre*^ mice exhibited impaired intestinal mucosal barrier function, disordered gut microbiota, and reduced SCFAs levels compared with aged control mice. Specifically, the relative abundance of *Akkermansiaceae* and *Barnesiellaceae* was significantly reduced in aged *TSP50* ^*fl/fl*^ *Nestin* ^*Cre*^ mice. *Akkermansiaceae* has previously been reported to be essential for promoting healthy aging, and *Akkermansiaceae* supplementation improves health‐span in naturally aging mice, while decreased abundance of *Barnesiellaceae* may be associated with cognitive impairment (Liu, Gao, et al. 2021; Ma et al. 2023). Notably, restoring gut microbiota balance and SCFAs production through FMT or TSP50 replenishment alleviated cognitive impairment and neuroinflammation in aged *TSP50* ^*fl/fl*^ *Nestin* ^*Cre*^ mice. Taken together, these results suggest that TSP50 may promote healthy aging by maintaining intestinal microecological balance and that SCFAs may be a key mediator through which TSP50 influences cognitive function and neuroinflammation.

Although our study demonstrates that TSP50 in NSCs plays a critical role in mitigating aging‐related cognitive decline and neuroinflammation by regulating the gut microbiota, the exact mechanisms remain unclear. Based on emerging literature and our findings, we propose several potential pathways. First, TSP50‐deficient NSCs may affect the gut microbiota by secreting signaling molecules—such as neurotransmitters, neurotrophic factors, or inflammatory cytokines—that influence intestinal barrier function and immune responses (Dicks 2022). Second, communication via the vagus nerve, a critical signaling pathway for bidirectional brain‐gut interaction, may be involved (Han et al. 2022). The absence of TSP50 in NSCs might disrupt vagus nerve‐mediated regulation of gut motility, mucosal immunity, and mucus secretion, indirectly contributing to microbiota dysbiosis. Finally, interactions within the neuro‐immune‐endocrine axis may serve as a key mediator. TSP50 deficiency in NSCs could activate the HPA axis, and HPA axis activation has been shown to be closely related to gut microbiota alterations (Rusch et al. 2023). Future studies should further explore these hypotheses, including analyses of secretory molecules from TSP50‐deficient NSCs and vagotomy experiments, to elucidate the specific mechanisms by which TSP50 in NSCs modulates the gut microbiota.

In addition, while our study highlights the association between reduced SCFAs levels and accelerated aging phenotypes in TSP50‐deficient mice, we recognize that direct evidence linking SCFAs supplementation to phenotypic rescue remains to be established. However, previous studies have robustly demonstrated the therapeutic potential of SCFAs in ameliorating neuroinflammation and cognitive decline. For instance, acetate and butyrate have been shown to suppress proinflammatory cytokines and microglial activation and improve cognitive performance in aging models (Ma et al. 2023; Matt et al. 2018; Zhou et al. 2022). Importantly, our findings align with these reports, as the reduced SCFAs levels in aged *TSP50* ^*fl/fl*^ *Nestin* ^*Cre*^ mice correlate with exacerbated neuroinflammation and cognitive decline, while restoration of SCFAs levels via FMT or TSP50 replenishment alleviated these phenotypes. These parallel outcomes strongly support the proposed mechanistic link between SCFAs and TSP50‐mediated effects. In future research, additional experiments involving exogenous SCFAs supplementation or receptor blockers will be essential to directly validate the role of SCFAs and elucidate their specific mechanisms.

Aging‐related cognitive decline is strongly associated with central inflammatory states (Li et al. 2023). In this study, we observed a significant increase in the number of astrocytes and microglia, as well as the levels of proinflammatory cytokines, in the brains of aged *TSP50* ^*fl/fl*^ *Nestin* ^*Cre*^ mice, suggesting that TSP50 deficiency can induce increased neuroinflammation. Previous studies have shown that gut microbiota can interact with the brain through systemic chronic inflammation to influence neuroinflammation, neurodegeneration, and aging (Mou et al. 2022). However, our study found that although FMT partially alleviated aging‐related cognitive impairment and neuroinflammation in aged *TSP50* ^*fl/fl*^ *Nestin* ^*Cre*^ mice, it did not completely restore these parameters to normal levels. Furthermore, enrichment analysis of differentially expressed genes in the hippocampus of mice showed that the loss of TSP50 may lead to the occurrence of neurodegenerative diseases, indicating that TSP50 may have a more direct regulatory role in central inflammation. This finding is significant because it highlights the potential of TSP50 as a therapeutic target for the treatment of aging‐related cognitive impairment, and we will continue this study in our future work.

In conclusion, our study identifies TSP50 as a key regulator of cognitive function and neuroinflammation during aging, with its effects mediated through the regulation of gut microbiota and SCFAs. This study reveals the mechanism of TSP50 in aging and provides new insights into the prevention and treatment of aging‐related cognitive impairment from the perspective of microbiota.

## Author Contributions

X.L. performed experiments, analyzed data, and wrote the manuscript. Y.C. helped with animal experiments. Z.G. helped analyze the data. X.L. helped in the generation and management of mice. Z.S. helped write the manuscript. F.G., S.W., C.Y., and Y.H. provided critical comments and suggestions. L.Z., G.W., and Y.S. provided technical help. J.L., X.Y., and Y.B. generated research funds, conceived the idea for the project, and led and coordinated the study. All authors have read and approved the manuscript.

## Ethics Statement

This study complies with all relevant ethical guidelines and institutional policies. Experimental protocols involving animals were approved by the Animal Advisory Committee of Northeast Normal University (NENU/IACUC, AP20191225). No human participants, human‐derived samples, or clinical trials were involved in this research.

## Conflicts of Interest

The authors declare no conflicts of interest.

## Supporting information


**Figure S1:** TSP50 deficiency in NSCs promotes intestinal barrier damage and systemic inflammation in aging mice.
**Figure S2:** Exacerbation of cognitive impairment and neuroinflammation in aged *TSP50* ^*fl/fl*^ *Nestin* ^*Cre*^ mice depends on the gut microbiota.
**Figure S3:** FMT alleviates cognitive dysfunction and neuroinflammation caused by TSP50 deficiency in aged mice.

## Data Availability

Raw 16S rRNA sequencing data and RNA sequencing data have been deposited in the National Center for Biotechnology Information (NCBI) Sequence Read Archive (SRA) database (https://www.ncbi.nlm.nih.gov/sra) with the accession numbers PRJNA1196995 and PRJNA1197079. All other raw data are available upon request from the corresponding authors.
