# Supplementary material for: TSP50 in Neural Stem Cells Regulates Aging‐Related Cognitive Decline and Neuroinflammation by Altering the Gut Microbiota
Source: Aging Cell. 2025 Aug 5;24(10):e70188. doi: 10.1111/acel.70188 (PMC12507395; doi:10.1111/acel.70188)
Supplement: Supplementary file 1 — Figure S1: TSP50 deficiency in NSCs promotes intestinal barrier damage and systemic inflammation in aging mice. Figure S2: Exacerbation of cognitive impairment and neuroinflammation in aged TSP50 fl/fl Nestin Cre mice depends on the gut microbiota. Figure S3: FMT alleviates cognitive dysfunction and neuroinflammation caused by TSP50 deficiency in aged mice. [file ACEL-24-e70188-s001.pdf]

Supplementary Information for

TSP50 in neural stem cells regulates aging-related cognitive decline and neuroinflammation by altering the gut microbiota

*Corresponding author: Jiawei Li, lijw447@nenu.edu.cn ; Xiaoguang Yang, yangxg168@nenu.edu.cn; Yongli Bao, baoyl800@nenu.edu.cn.

This word file includes:

Supplementary Figure 1 to 3

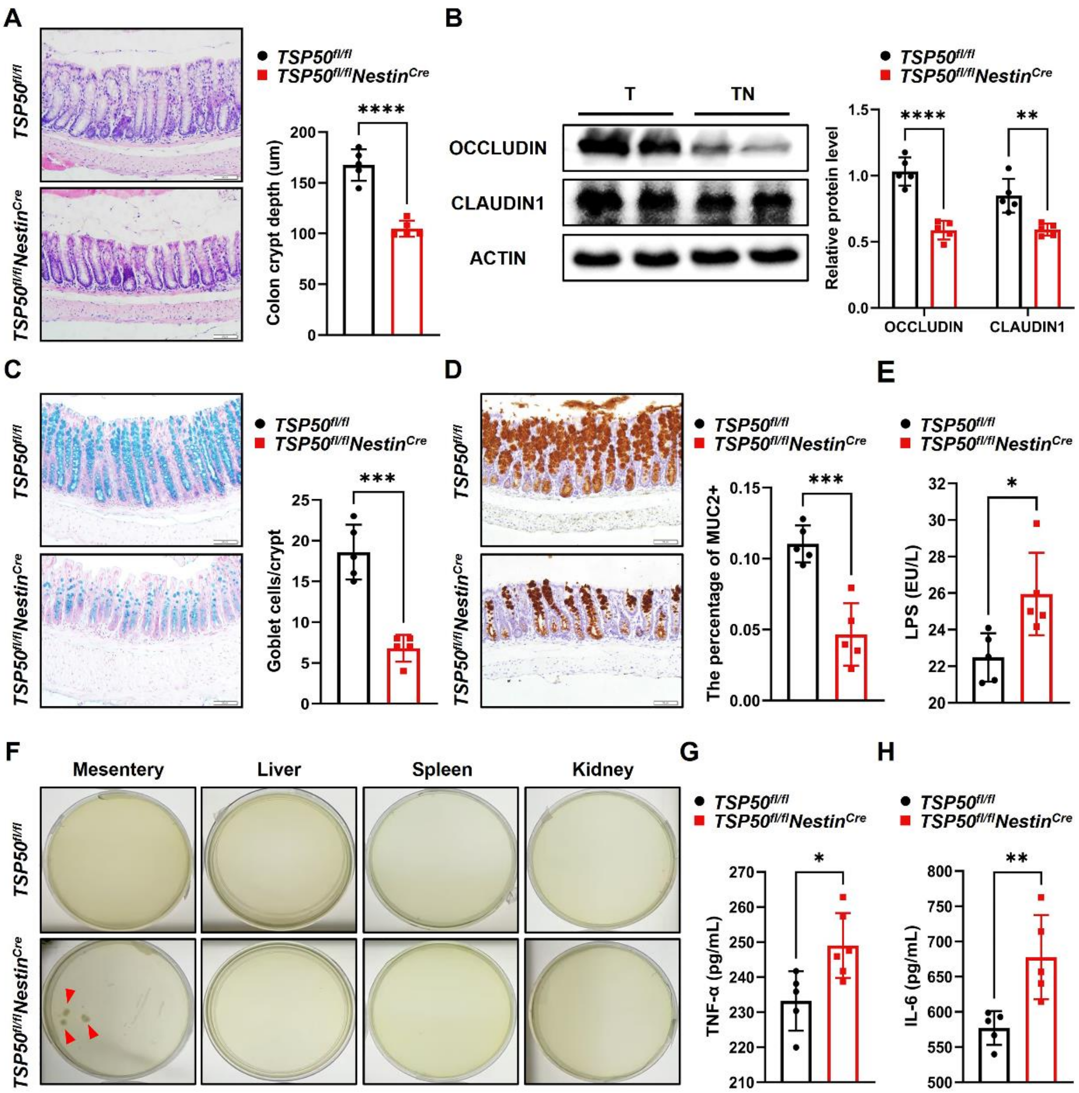

Supplementary Figure 1: TSP50 deficiency in NSCs promotes intestinal barrier damage and systemic inflammation in aging mice.

(A) Representative HE-stained images of colon tissues from aged mice and quantitative analysis of crypt depth. Scale bar: 50 µm. (B) Western blot analysis and quantification of Occludin and Claudin1 protein levels in the colon of aged mice. (T: *TSP50*$^{fl/fl}$; TN: *TSP50*$^{fl/fl}$*Nestin*^{Cre}) (C) Representative images of Alcian blue staining of colon tissues from aged mice and quantitative analysis. Scale bar: 50 µm. (D) Representative images of IHC staining of MUC2 expression in the colon of aged mice and quantitative analysis. Scale bar: 50 µm. (E) ELISA analysis of serum LPS levels in aged mice. (F) Bacterial translocation in mesentery, liver, spleen, and kidney of aged mice. (G) ELISA analysis of serum TNF-α levels in aged mice. (H) ELISA analysis of serum IL-6 levels in aged mice. Values are expressed as means ± SEM, * $P < 0.05$, ** $P < 0.01$, *** $P < 0.001$, **** $P < 0.0001$.

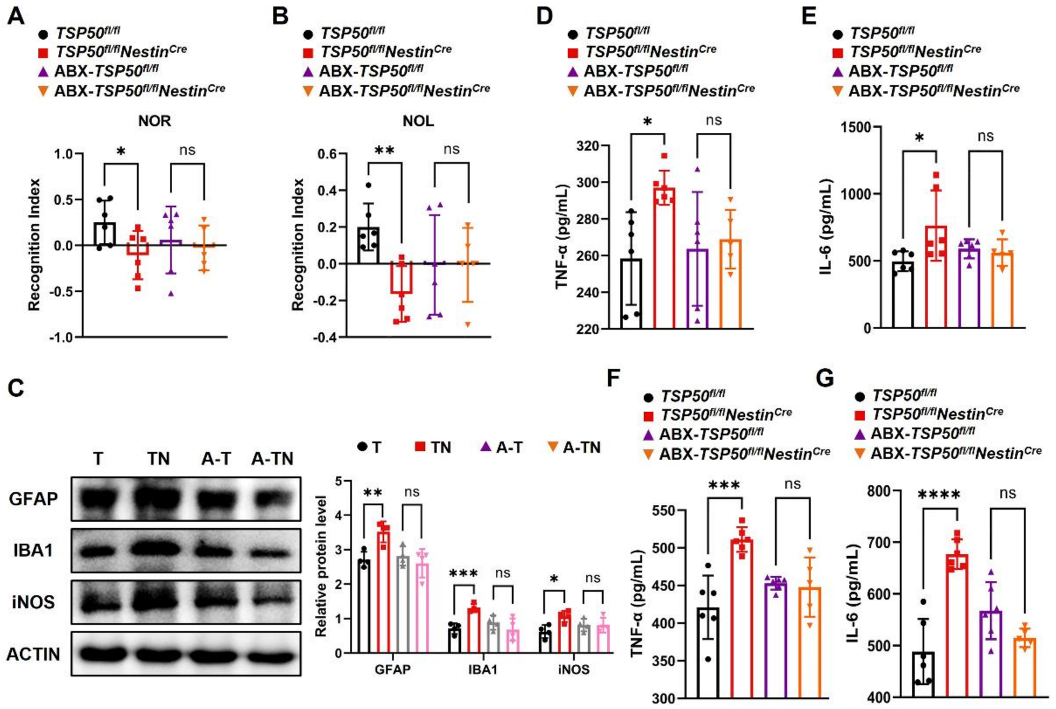

Supplementary Figure 2: Exacerbation of cognitive impairment and neuroinflammation in aged *TSP50*$^{fl/fl}$*Nestin*^{Cre} mice depends on the gut microbiota

(A) NOR test results of aged mice after ABX. (B) NOL test results of aged mice after ABX. (C) Western blot analysis and quantification of GFAP, IBA1, and iNOS protein levels in the hippocampus of aged mice after ABX. (T: *TSP50*$^{fl/fl}$; TN: *TSP50*$^{fl/fl}$*Nestin*^{Cre}; A-T: ABX-*TSP50*$^{fl/fl}$; A-TN: ABX-*TSP50*$^{fl/fl}$*Nestin*^{Cre}) (D) ELISA analysis of serum TNF-α levels in aged mice after ABX. (E) ELISA analysis of serum IL-6 levels in aged mice after ABX. (F) ELISA analysis of hippocampal TNF-α levels in aged mice after ABX. (G) ELISA analysis of hippocampal IL-6 levels in aged mice after ABX. Values are expressed as means ± SEM, ns (no significance), * $P < 0.05$, ** $P < 0.01$, *** $P < 0.001$, **** $P < 0.0001$.

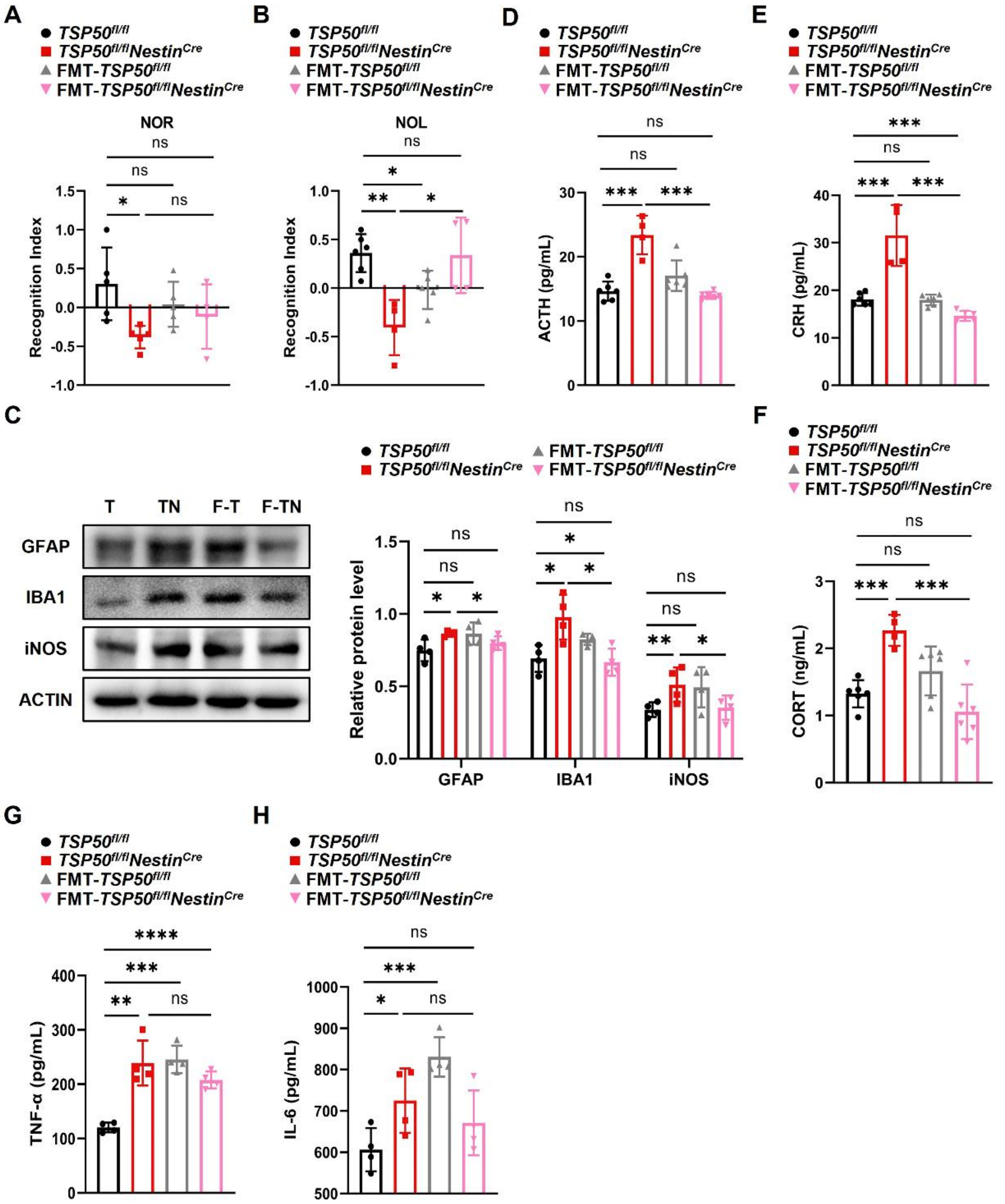

Supplementary Figure 3: FMT alleviates cognitive dysfunction and neuroinflammation caused by TSP50 deficiency in aged mice.

(A) NOR test results of aged mice after FMT. (B) NOL test results of aged mice after FMT. (C) Western blot analysis and quantification of GFAP, IBA1, and iNOS protein levels in the hippocampus of aged mice after FMT. (T: *TSP50*$^{fl/fl}$; TN: *TSP50*$^{fl/fl}$*Nestin*^{Cre}; F-T: FMT-*TSP50*$^{fl/fl}$; F-TN: FMT-*TSP50*$^{fl/fl}$*Nestin*^{Cre}) (D) ELISA analysis of serum ACTH levels in aged mice after FMT. (E) ELISA analysis of serum CRH levels in aged mice after FMT. (F) ELISA analysis of serum CORT levels in aged mice after FMT. (G) ELISA analysis of serum TNF-α levels in aged mice after

FMT. (H) ELISA analysis of serum IL-6 levels in aged mice after FMT. Values are expressed as means ± SEM, ns (no significance), * $P < 0.05$, ** $P < 0.01$, *** $P < 0.001$, **** $P < 0.0001$.
